# Psoas Muscle Area Measured with Computed Tomography at Admission to Intensive Care Unit: Prediction of In-Hospital Mortality in Patients with Pulmonary Embolism

**DOI:** 10.1155/2020/1586707

**Published:** 2020-03-07

**Authors:** Ibrahim Akkoc, Mehmet Toptas, Mazhar Yalcin, Eren Demir, Yasar Toptas

**Affiliations:** ^1^Departments of Anesthesiology and Reanimation, Haseki Training and Research Hospital, University of Health Sciences, Istanbul, Turkey; ^2^Departments of Radiology, Haseki Training and Research Hospital, University of Health Sciences, Istanbul, Turkey; ^3^Department of Anesthesiology and Reanimation, Sakarya University Faculty of Medicine, Sakarya, Turkey

## Abstract

**Aim:**

Sarcopenia, a core component of physical frailty, is an independent risk factor for suboptimal health outcomes in hospitalized patients, especially in the intensive care patients. Psoas muscle areas can be assessed to identify sarcopenia. The aim of this study was to determine the prognostic value of psoas muscle area measured with CT for the prediction of in-hospital mortality in patients with pulmonary embolism at admission to the intensive care unit.

**Methods:**

Patients with an admission abdominal computed tomography scan and requiring intensive care unit (ICU) stay were reviewed. Selected clinical data of patients admitted to intensive care unit for the management of pulmonary embolism were collected. Using CT scan images at the level of L3 vertebra, the psoas muscle area value was obtained by dividing the sum of the right and left psoas muscle areas into the body surface area.

**Results:**

In-hospital mortality rate was 22.5% in 89 patients. The pulmonary embolism patients with in-hospital mortality had higher PESI and lower value of psoas muscle area, in addition to the lower systolic blood pressure and arterial oxygen saturation at admission. The increase in the value of psoas muscle area is associated with a decrease in the rate of in-hospital mortality. In patients with in-hospital mortality related to pulmonary embolism, the higher PESI and the lower value of psoas muscle area were considered in accordance with the outcome of patients.

**Conclusions:**

For the prediction of in-hospital mortality risk in patients with pulmonary embolism managed in intensive care unit, the psoas muscle area value has a merit to be used among the routine diagnostic procedures after further studies conducted with different severity of pulmonary embolism.

## 1. Introduction

Acute pulmonary embolism presents on a spectrum of severity with the most severe presentations carrying a substantial risk of morbidity and mortality. There is also continuing attempts for rapid and proper evaluation of patients with PE, formulation of a treatment plan, and mobilization of the necessary resources to provide the highest level of care to reduce mortality and morbidity with long-term effects. Major underlying conditions (cancer and cardiac or respiratory disease), clinical signs of right ventricular dysfunction (tachycardia and hypotension) and hypoxemia, and frailty in the elderly are the main clinical determinants of the outcome of patients with pulmonary embolism. Risk management poses a significant challenge when facing complex decisions regarding potential use of invasive procedures in older patients in the management of pulmonary embolism [1–4].

The cornerstone of appropriate management in patients with pulmonary embolism in the intensive care unit is early detection of mortality risk. For this purpose, the applications of several tools are used at admission and during follow-up. There is a need for new prognostic indicators with satisfactory sensitivity and specificity in order to achieve early detection of mortality risk in patients managed for pulmonary embolism in intensive care unit. To improve critical care of these patients, one of the main arms of studies needs to perform new investigations that make possible the further refinement of frailty assessment tools to facilitate enhanced shared decision-making between aging patients and their physicians.

Frailty is difficult to diagnose, particularly within intensive care settings, due to its coexistence with other age-related conditions and because of the lack of a universally accepted clinical definition [5]. Sarcopenia, or age-related loss of skeletal muscle and muscle strength, is a key physical component of frailty. Decline in skeletal muscle function and mass are consequences of age-related deterioration in the function of several physiological systems including endocrine, neurological, cardiovascular, and/or immunological dysfunctions [6, 7]. Diagnosis of sarcopenia is based on the knowledge obtained by low levels of measures for three parameters: muscle strength, quantity/quality, and physical performance as an indicator of severity [8]. Nevertheless, its definition remains a matter of discussion and there is no globally accepted consensus for its diagnosis [9].

For the diagnosis of sarcopenia, there are many methods to evaluate muscle mass, including anthropometry, bioelectrical impedance analysis, and medical imaging. The common imaging modalities for evaluating muscle mass include whole-body dual-energy X-ray absorptiometry, CT, and MRI. Medical imaging is useful for tracking longitudinal changes during the follow-up of patients with sarcopenia [10]. Despite these efforts, radiation exposure is still high, which limits the use of CT for body composition assessment only. Although the assessment of sarcopenia based on whole body imaging is the most accurate, it is very time consuming and costly; thus, it may not be practical in most clinical settings. Therefore, identifying which anatomical level or muscles best represents the total lean body mass is a very important issue. To date, there have been three main types of measurement: total abdominal muscle area at the lumbar spine level, psoas muscle area in the lumbar spine level, and thigh muscles at the mid-thigh level. It is possible to determine psoas muscle area when there are previously performed CT scans acquired during the diagnosis/treatment/follow-up procedures [10]. The aim of this study was to determine prognostic value of computed tomography-based psoas muscle area measured at admission to the intensive care unit for the prediction of mortality in patients with pulmonary embolism.

## 2. Materials and Methods

This retrospective study was conducted at the Haseki Training and Research Hospital with 89 adult patients who were treated at the intensive care unit with diagnosis of pulmonary embolism by computed tomography. The approval of Human Research Ethics Committee of our institution was obtained. After informed consent of patients or relatives, from the electronic hospital records of patients, selected clinical variables of the patients at admission and during follow-up were recorded. The pulmonary embolism severity index (PESI) was used to assess the clinical severity of the patients in accordance with the ESC guideline [11, 12].

### 2.1. Psoas Area Measurement

**Figure 1:**
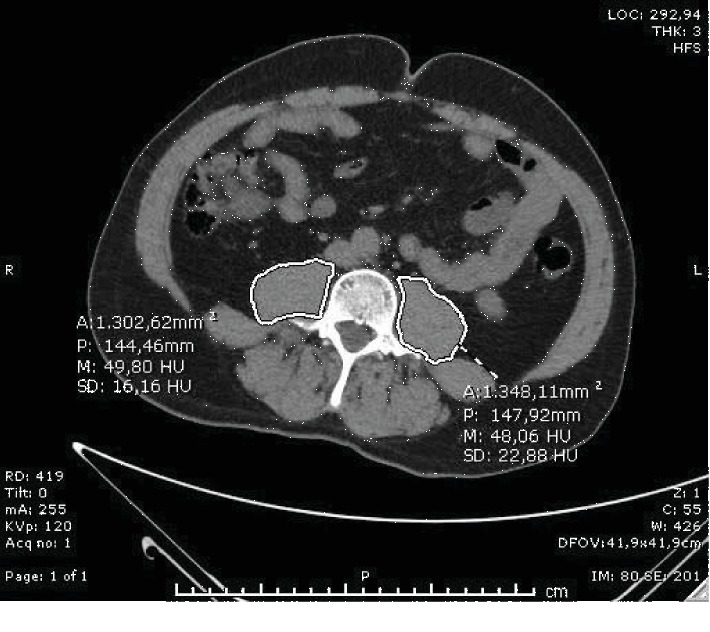
43-year-old man with pulmonary embolism managed in intensive care unit. The value of psoas muscle area was obtained by dividing the sum of the right and left psoas muscle areas at the level of L3 vertebrae into body surface areas using single axial CT scan images. He had lower PESI scores, high arterial oxygen saturation, and lower respiratory rate without in-hospital mortality.

**Figure 2:**
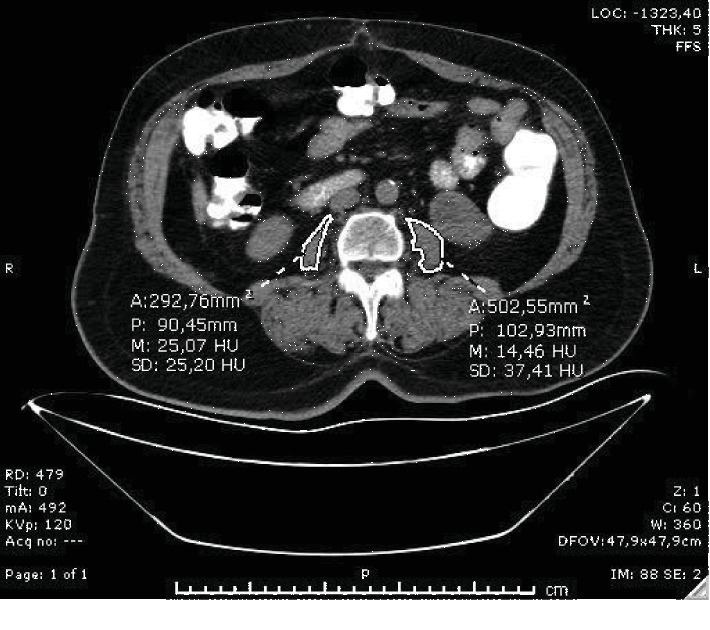
65-year-old woman with pulmonary embolism. The value of psoas muscle area was obtained by dividing the sum of the right and left psoas muscle areas at the level of L3 vertebrae into body surface areas using single axial CT scan images. He had higher PESI scores, lower arterial oxygen saturation, and higher respiratory rate in-hospital mortality.

A radiologist who was blinded to patient outcomes performed quantitative assessment of psoas muscle areas using the available CT scan images at the caudal end of L3 vertebra. Three measurements were taken of the left and right psoas and their average used for analysis. The value of psoas muscle area was obtained by dividing the sum of the right and left psoas muscle areas into the body surface area for normalization (Figures 1 and 2).

### 2.2. Statistical Analysis

The SPSS software (IBM SPSS, Version 22.0, IBM Corporation, Armonk, NY, USA) was used for the statistical analyses on the data. Normality was evaluated by the Shapiro-Wilk statistics. Continuous data are presented as mean with standard deviation and analyzed with *t* test. Categorical data are reported as proportions and, where appropriate, tested for significance using a chi-square test. Pearson correlation analysis was performed to examine associations of in-hospital mortality with clinical parameters. All statistical tests were two-tailed, and *p* < 0.05 was set as the statistical significance level.

## 3. Results

There was no significant difference between the pulmonary embolism patients with or without in-hospital mortality with regard to the age and gender of patients. Considering clinical variables, in the pulmonary embolism patients with in-hospital mortality compared to without in-hospital mortality, the values of systolic blood pressure and arterial oxygen saturation at admission were significantly higher but the values of heart and respiratory rates at admission were significantly lower (*p* < 0.05); however, the values of body temperature at admission and rates of fibrinolytic treatment and mechanical ventilation support were found similar in the pulmonary embolism patients with or without in-hospital mortality (*p* > 0.05) (Table 1). With regard to laboratory findings, no significant difference was obtained with regard to the values of white blood cell, platelet, D-dimer, and troponin values at admission in the pulmonary embolism patients with or without in-hospital mortality (*p* > 0.05).

To examine the role of clinical factors producing significant difference in the in-hospital mortality rate of patients with pulmonary embolism with logistic regression analysis (Table 2), the results showed that only the value of psoas muscle area significantly reduced the rate of in-hospital mortality with an odds ratio of 0.259 (*p* < 0.05).

**Figure 3:**
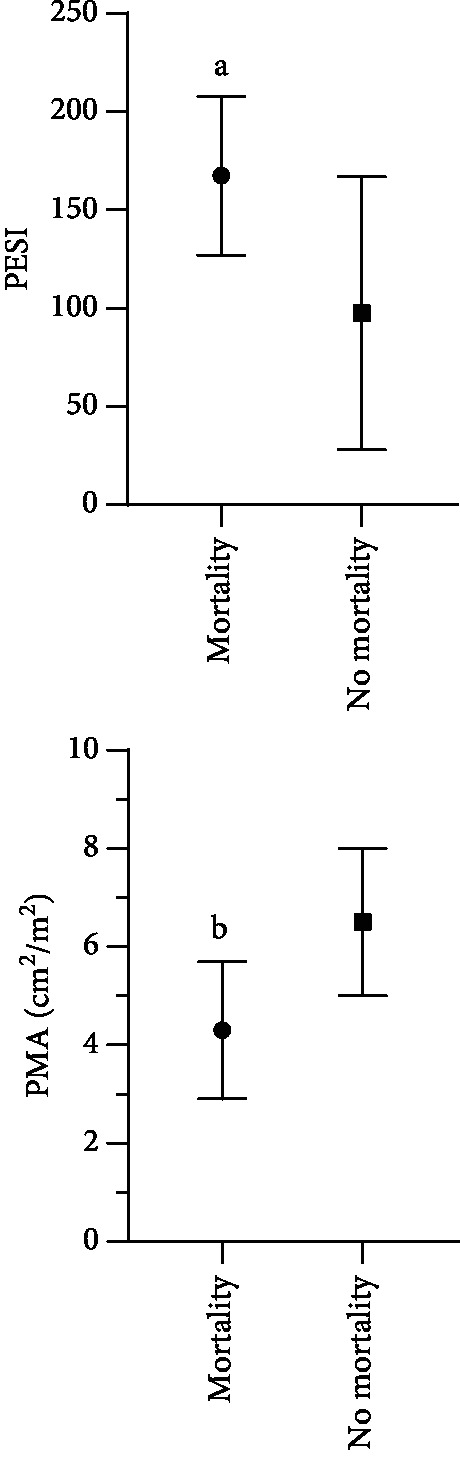
Pulmonary embolism severity index (PESI) and value of psoas muscle area (PMA) with regard to in-hospital mortality in patients with pulmonary embolism managed in intensive care unit. Data were presented as mean with standard deviation. ^a,b^*p* < 0.05 vs. pulmonary embolism patients with no in-hospital mortality.

As presented in Figure 3, the PESI of pulmonary embolism patients with in-hospital mortality was significantly higher than that of the pulmonary embolism patients without in-hospital mortality (*p* < 0.05). The value of psoas muscle area in the pulmonary embolism patients with in-hospital mortality was significantly lower than that in the pulmonary embolism patients without in-hospital mortality (*p* < 0.05).

## 4. Discussion

In the current retrospective study of pulmonary embolism patients with a considerable comparable clinical and laboratory background, the place of using the value of psoas muscle area to predict the in-hospital mortality was assessed, as an easy procedure when there is available abdominal CT scan previously obtained. The findings supported that the pulmonary embolism patients with in-hospital mortality had higher PESI and lower value of psoas muscle area, in addition to the lower systolic blood pressure and arterial oxygen saturation at admission. The increase in the value of psoas muscle area is associated with a decrease in the rate of in-hospital mortality. In patients with in-hospital mortality related to pulmonary embolism, the higher PESI and the lower value of psoas muscle area were considered in accordance with the outcome of patients.

The findings of the current study also present the importance of follow-up of these patients with pulmonary embolism after discharge from intensive care. With a comprehensive care plan for their sarcopenia and physical frailty, physicians need to pay attention to reasonable polypharmacy, the management of sarcopenia, the treatable causes of weight loss, and other components of frailty.

Pulmonary embolism (PE) is characterized by embolic occlusion of one or more pulmonary arteries. The incidence of symptomatic PE is estimated to be about 0.5–1 per 1000 people per year, and is increasing as the population ages. The clinical manifestations of PE range from no symptoms to sudden death, depending on the degree of obstruction of the pulmonary vasculature and the cardiovascular reserve of the patient. Hospitalization is considered in patients with a high PESI score, additionally with either hemodynamic instability, need for oxygen or parenteral analgesics, or comorbidities [13, 14].

Sarcopenia and frailty are important conditions that become increasingly prevalent with age. Since there is considerable overlapping between sarcopenia and physical frailty, there is a need to think these as a whole because of their close relationship with the aging. In recent years, numerous studies have shown the importance of the patient's body composition including muscle mass as a prognostic factor for clinical outcome in various settings. It therefore correlates in some extent with patient's general physical condition and frailty [15]. Diagnostic criteria are clearly essential for the recognition of sarcopenia and frailty in clinical practice.

Sarcopenia gradually progress with age; hence, it is necessary to assess sarcopenia to predict outcomes in elderly patients with pulmonary disorders including pulmonary embolism [16–18]. The current definition of sarcopenia is based on quantitative (muscular mass) and functional (muscular strength and function) criteria. Currently, there is no single technique available that can measure all these criteria in the same settings. The first step in defining a patient with sarcopenia is quantitative and consists in quantifying the skeletal muscular mass. This quantitative evaluation is accomplished by different techniques, of which imaging has a major part [19]. CT provides excellent images of muscles and has the advantage of being more available than other imaging modalities.

Frailty is defined as a clinically recognizable state of increased vulnerability, resulting from aging-associated decline in reserve and function across multiple physiologic systems [20–23]. In the absence of a gold standard, frailty has been operationally defined by Fried et al. [22] as a condition meeting 3 of the 5 phenotypic criteria indicating compromised energetics, namely, low grip strength, low energy, slowed waking speed, low physical activity, and unintentional weight loss [21].

Frailty, one of the most important health problems in the older adult population (≥65 years of age), may present with a high prevalence of 38%. There are also new studies trying to identify frailty in younger patients. The rate of frailty in adults aged ≥40 years can be 16% [24]. The overall prevalence of frailty in adults age 65 years and older has been estimated at approximately 10% [25]. Frailty has been correlated with increased morbidity and mortality and decreased functional status in patients undergoing clinical and intensive care procedures.

There is lack of corresponding consensus despite the wide range of tools that are available to diagnose frailty and its dimensions in clinical practice [26, 27]. Thus, the tool needs to be validated and simple to use, and provide results that is easy to interpret for appropriately guiding goal setting and care planning such that identification of frailty is able to meaningfully impact the management of the individual in a contextual and appropriate way [27].

Measurements of psoas muscle area over time were essential to identify muscle loss or sarcopenia. A single time point measurement could not detect change and was thus unable to predict survival [28]. Physicians need to keep in mind that the reduced psoas muscle area may not be a representative of total muscle area change and it should not be used to substitute total skeletal muscle as reported by a study performed to predict survival in patients with ovarian cancer [28].

Body mass index less than 20 kg/m2 is a parameter that is commonly used to screen nutritional status and calculate nutrient requirements, in addition to hypoalbuminemia. However, it fails to account for this large cohort of patients with low muscularity who may benefit from more aggressive and integrated nutritional and rehabilitative strategies during management in the intensive care unit to attenuate additional muscle loss in patients who already have sarcopenia [29]. Moisey et al. [29] noted that sarcopenia is highly prevalent in the elderly with traumatic injuries and adequate muscle mass increases ventilator-free days, ICU-free days, and mortality in elderly patients stayed in the intensive care unit.

Huber et al. [30] retrospectively reviewed the records of patients who underwent endovascular aneurysm repair to determine the predictivity of psoas muscle area for postoperative mortality. They used preprocedure CT scans to measure the psoas muscle area of patients. They found that the measurement of psoas muscle area is more predictive for mortality than other clinical parameters. Hawkins et al. [31] determined the place of psoas muscle size as a finding of frailty for the prediction of risk-adjusted outcomes in moderate to high-risk patients after aortic valve placement. They calculated the psoas index by dividing the mean psoas cross-sectional area to the body surface area. They noted that psoas index is an easily obtained and reproducible measure of frailty that predicts risk-adjusted resource utilization, morbidity, and long-term mortality. Toptas et al. [32] assessed the impact of sarcopenia on the mortality of patients managed in the intensive care unit. Their indications of admission to the intensive care unit were classified as urgent, internal, or surgical disorders. In that study, the measurements of left and right psoas muscle areas were performed to obtain the total psoas area at the level of L3 lumbar vertebra on CT images. They suggested that when there is available abdominal CT scanning of patients, the measurement of total psoas area could be used as an indicator of increased mortality in patients managed in the intensive care unit. Jones et al. [33] found that sarcopenia, based on the measurements of psoas muscle areas at the level of third vertebra, may be used as a frailty marker, for the prediction of major complications.

This study was performed retrospectively with a cohort of patients who underwent abdominal CT scan previously performed within the last 30 days. There are several limitations to this study including its single center and retrospective nature that inherently produces some element of selection bias. In this study, evaluating clinical information found in the charts of patients, we could consistently find the PESI score but not the calculated ratio of cardiac ventricles, which was measured from their CT of chest and used during diagnostic workup. If present, this finding could be helpful to better understand the severity of pulmonary embolism. Patients' height and weight data only being known for within one month of the date of the CT scan, no knowledge surrounding the hydration status of the patient at the time of their CT scan and no data regarding the stability of patients' nutritional state, although these were factors that can affect the measurements. In addition, the presence of spinal pathology or chronic lower back pain can change psoas muscle structure. The generalizability of the results is also limited by the changing prevalence of frailty according to age of populations. Psoas area measurement, while an alternative measure of sarcopenia that can be correlated with many different measures of sarcopenia, is not a comprehensive frailty measure. This limited sarcopenia assessment includes an inherent limitation of any simple and fast measurement.

Collectively, the current literature and findings of this study allow us to conclude that assessment of sarcopenia with psoas muscle area is a potential prognostic tool during diagnosis and management of pulmonary embolism in especially older patients. Our study suggests that in elderly patients with pulmonary embolism, sarcopenia and physical frailty assessment needs to be integrated into the currently existing management strategies to better appraise the mortality risk. The identification of sarcopenia and physical frailty helps physicians to applicate appropriate therapies and help patients make properly informed choices. Since there is currently little evidence that sarcopenia and physical frailty-based management could improve outcomes of elderly patients with pulmonary embolism, it is necessary to conduct further studies to establish a good relationship of tools for sarcopenia and physical frailty evaluation with status, prognosis, and mortality of patients with pulmonary embolism managed in the intensive care unit.

## Figures and Tables

**Table 1:** Selected demographic, clinical, and laboratory parameters obtained at admission in patients with pulmonary embolism managed in intensive care unit with regard to in-hospital mortality.

	In-hospital mortality (*n* = 20)	No in-hospital mortality (*n* = 69)	Significance
Age (y)	70.7 ± 12.7	66.5 ± 16.2	NS
Gender (male) (*n*, %)	15 (75.0%)	36 (52.2%)	NS
Systolic blood pressure at admission (mmHg)	80 ± 12.1	102.3 ± 23.1	*p* < 0.05
Heart rate at admission (pulse/min)	117.5 ± 6.4	106.5 ± 17.6	*p* < 0.05
Respiratory rate at admission (min)	27.2 ± 1.9	22.1 ± 4.1	*p* < 0.05
Body temperature at admission (°C)	36.6 ± 0.5	36.6 ± 0.8	NS
Arterial oxygen saturation at admission (%)	81.8 ± 6.1	88.1 ± 6.9	*p* < 0.05
Fibrinolytic treatment (*n*, %)	4 (20.0%)	27 (%39.1)	NS
Mechanical ventilation support (*n*, %)	7 (35.0%)	14 (20.3%)	NS
White blood cell at admission (mm^3^)	10,545 ± 4277	9309 ± 3428	NS
Platelet at admission (×10^9^/L)	278 ± 122	298 ± 102	NS
D-dimer at admission (ng/mL)	6415 ± 1440	8783 ± 2100	NS
Troponin at admission (ng/mL)	2.9 ± 1.2	2.9 ± 2.1	NS

Data were expressed as mean with standard deviation or number (%). They were analyzed with *t* and chi-square tests as appropriate. They were expressed as mean ± standard deviation. NS: not significant.

**Table 2:** Results of logistic regression analysis presenting odds of pulmonary embolism severity index (PESI), heart rate, respiratory rate, arterial oxygen saturation, systolic blood pressure, and value of psoas muscle area with regard to in-hospital mortality in patients with pulmonary embolism managed in intensive care unit.

	Odds ratio (95% confidence interval)	Significance
PESI	1.001 (0.970-1.034)	NS
Heart rate (pulse/min)	0.930 (0.787-1.099)	NS
Respiratory rate (min)	0.173 (0.101-0.296)	NS
Arterial oxygen saturation (%)	0.948 (0.806-1.115)	NS
SBP (mmHg)	0.958 (0.875-1.051)	NS
Psoas muscle area (cm^2^/m^2^)^∗^	0.259 (0.122-0.551)	*p* < 0.05

^∗^Psoas muscle area meaningfully reduced in-hospital mortality rate with an odds ratio of 0.259.

## Data Availability

The data used to support the findings of this study are available from the corresponding author upon request.
